# Analysis of the learning curve for transvaginal natural orifice transluminal endoscopic surgery in ovarian cystectomy

**DOI:** 10.3389/fmed.2025.1629418

**Published:** 2025-06-13

**Authors:** Qiang Zhang, Wenting Dong, Biao Huang, Aijie Xie, Zhaolin Gong, Dan Feng, Li He, Yonghong Lin

**Affiliations:** ^1^Department of Gynecology and Obstetrics, Chengdu Women’s and Children’s Central Hospital, School of Medicine, University of Electronic Science and Technology of China, Chengdu, China; ^2^Sichuan Jinxin Xinnan Women & Children Hospital, Chengdu, China; ^3^Department of Obstetrics and Gynecology, Women and Children's Hospital of Chongqing Medical University, Chongqing, China; ^4^NHC Key Laboratory of Birth Defects and Reproductive Health, Chongqing, China

**Keywords:** transvaginal natural orifice transluminal endoscopic surgery, ovarian cystectomy, learning curve, CUSUM analysis, minimally invasive surgery

## Abstract

**Objective:**

This study aimed to evaluate the learning curve for transvaginal natural orifice transluminal endoscopic surgery (vNOTES) in ovarian cystectomy and to identify perioperative factors influencing operative time.

**Methods:**

This prospective observational study included 39 patients who underwent vNOTES ovarian cystectomy at Chengdu Women’s and Children’s Central Hospital between June 2022 and June 2024. Patients were grouped into two surgical phases based on the operating team’s self-assessed proficiency. Cumulative sum analysis of operative time (CUSUMOT) was used to model the learning curve and define distinct learning stages. Multivariate linear regression was performed to identify independent predictors of operative time.

**Results:**

The mean patient age was 35.14 ± 9.73 years, and the mean operative time was 74.01 ± 30.09 min. Three cases (7.7%) required intraoperative conversion to transumbilical laparoscopy, and two patients (5.1%) experienced perioperative complications. CUSUMOT analysis revealed four distinct learning phases: learning (9 cases), plateau (10 cases), challenging (12 cases), and mature (8 cases). Operative time during the mature phase was significantly shorter than in earlier phases. Multivariate regression identified pelvic adhesions (*β* = 6.92, *p* = 0.027), bilateral cysts (β = 6.38, *p* = 0.019), cyst diameter (β = 2.85 per cm, *p* = 0.026), and learning curve phase (*β* = −17.10 for Phase II, *p* = 0.035) as independent predictors of operative time.

**Conclusion:**

vNOTES is a safe and feasible approach for ovarian cystectomy with a measurable learning curve. Proficiency can be achieved after approximately 20 cases. Pelvic adhesions, cyst characteristics, and surgical experience significantly impact operative time. CUSUM analysis is a useful tool for evaluating surgical competency and guiding clinical training in vNOTES procedures.

## Background

Ovarian cysts are common gynecological conditions encountered in women of reproductive age. While many cysts are benign and asymptomatic, some may cause pelvic pain, menstrual irregularities, or adnexal torsion and thus require surgical intervention (1, 2). Traditionally, laparoscopic ovarian cystectomy via transabdominal access has been the standard minimally invasive approach. However, the evolution of minimally invasive gynecologic surgery has led to the emergence of transvaginal natural orifice transluminal endoscopic surgery (vNOTES), which utilizes the natural vaginal route to access the peritoneal cavity and perform various surgical procedures (3, 4).

Compared to conventional laparoscopy, vNOTES has several potential advantages, including the avoidance of visible abdominal scars, reduced postoperative pain, faster recovery, and shorter hospital stay (5, 6). Despite these benefits, the widespread adoption of vNOTES has been limited by the technical challenges associated with the procedure and the steep learning curve for gynecologic surgeons, particularly in ovarian surgery where anatomical complexity and pelvic adhesions are common (7, 8).

Previous studies on vNOTES have mainly focused on its application in hysterectomy and adnexectomy, with limited data available regarding its use in ovarian cystectomy (9, 10). Furthermore, the learning process for vNOTES ovarian cystectomy, including how proficiency develops over time and which perioperative factors influence surgical performance, remains poorly understood.

This study aimed to analyze the learning curve for vNOTES ovarian cystectomy by evaluating operative time and cumulative sum (CUSUMOT) analysis across consecutive cases (11). Additionally, we sought to identify perioperative factors influencing operative time and surgical outcomes, thereby providing evidence to guide the safe and effective implementation of vNOTES in ovarian surgery.

## Methods

### Study design and participants

This prospective observational study was conducted at Chengdu Women’s and Children’s Central Hospital between June 2022 and June 2024. The study protocol was approved by the hospital’s Ethics Committee (Approval No. 2022207) and registered with the Chinese Clinical Trial Registry (ChiCTR2200059282). Inclusion criteria were as follows: female patients aged 18–60 years; patients with ovarian cysts larger than 5 cm in maximum diameter, as confirmed by imaging and physical examination, with surgical indications (i.e., pathological or symptomatic physiological cysts), and who consented to surgery; and patients who voluntarily agreed to participate in the study and signed informed consent. Exclusion criteria were as follows: no history of sexual activity; suspected malignancy based on imaging or laboratory findings; a clear history of severe pelvic adhesions as confirmed by prior surgery or strongly suspected on gynecological examination; and active vaginal infection.

### Surgical phases and grouping

Patients were sequentially allocated into two phases based on the surgical team’s experience. Phase I included patients with relatively simple preoperative evaluations based on factors such as prior pelvic surgery, cyst type, and cyst size. After the surgical team determined that they had achieved initial technical proficiency in vNOTES ovarian cystectomy, Phase II commenced. In Phase II, patients were included solely based on the study’s inclusion and exclusion criteria, regardless of case complexity. The study concluded once the surgical team considered the technique to be fully mastered.

### Data collection

Data on baseline characteristics and perioperative outcomes were collected, including age, height, weight, length of hospital stay, surgical and obstetric history, maximum cyst diameter, cyst type (endometriotic vs. non-endometriotic), laterality (unilateral vs. bilateral), operative time, intraoperative conversion, estimated blood loss, and postoperative complications.

### Surgical procedure

All surgeries were performed under general anesthesia with the patient in the lithotomy position. A 1.5-cm incision was made in the posterior vaginal fornix, and a single-use access port (HK-TH-60.4TY, Beijing Aerospace Cardiotechnology Institute) was inserted. Pneumoperitoneum was established using CO₂, followed by the insertion of a 10-mm 30° rigid laparoscope (Karl Storz, Germany). After bowel retraction, the ovarian cyst was dissected from surrounding structures. An incision was made along the long axis of the cyst, and the cyst wall was separated from the ovarian cortex. Following cystectomy, the remaining ovarian tissue was sutured using absorbable sutures to perform ovarian reconstruction. The pelvic cavity was irrigated, and the vaginal incision was closed with 2–0 barbed sutures.

### Surgical team composition and training

All vNOTES ovarian cystectomies were performed by a dedicated gynecologic surgical team. The lead surgeon was an attending gynecologist with over 10 years of experience in laparoscopic surgery. Two attending gynecologists served as consistent first assistants throughout the study. During the initial learning phase, the procedures were performed under the direct supervision of an experienced vNOTES surgeon who had independently completed over 50 vNOTES cases. Once the lead surgeon demonstrated the ability to perform the surgery independently, the mentor was available on-call and only participated intraoperatively if required. The nursing staff included circulating and scrub nurses with laparoscopic experience, assigned based on standard hospital scheduling protocols.

### Learning curve analysis

To evaluate the learning curve of vNOTES ovarian cystectomy, a two-dimensional analysis was performed. The *X*-axis represented the chronological sequence of surgeries, while the *Y*-axis represented the cumulative sum of deviations from the mean operative time. The CUSUMOT for each case was calculated using the formula:

CUSUMOT=∑(OTi−μ)

where OTᵢ represents the operative time of the ith case, and μ is the mean operative time of all cases. For example, CUSUMOT₁ = OT₁ − μ; CUSUMOT₂ = CUSUMOT₁ + (OT₂ − μ), and so forth. An upward trend in the CUSUMOT curve indicated a learning phase, while a downward trend suggested a transition toward surgical maturity.

### Sample size estimation and statistical assumptions

As this was an exploratory prospective observational study focused on delineating the learning curve of vNOTES ovarian cystectomy using CUSUM (cumulative sum) analysis, formal *a priori* sample size calculation was not performed. Instead, the study aimed to include a minimum of 30 consecutive cases based on previous literature suggesting that 20–30 procedures are generally required to reach technical proficiency in similar minimally invasive gynecologic surgeries (12, 13). Ultimately, 39 cases were enrolled to ensure adequate representation across the anticipated learning phases. The primary analysis was descriptive and hypothesis-generating, rather than designed to test a single pre-specified hypothesis.

### Statistical analysis

All statistical analyses were conducted using SPSS version 26.0 (IBM Corp., Armonk, NY). Continuous variables were expressed as means ± standard deviations and compared using Student’s *t*-test or Welch’s *t*-test, as appropriate. Categorical variables were presented as counts and percentages and analyzed using the chi-square test or Fisher’s exact test. Multiple linear regression analysis was performed to identify factors independently associated with operative time. All tests were two-sided, and a *p*-value <0.05 was considered statistically significant.

## Results

**Figure 1:**
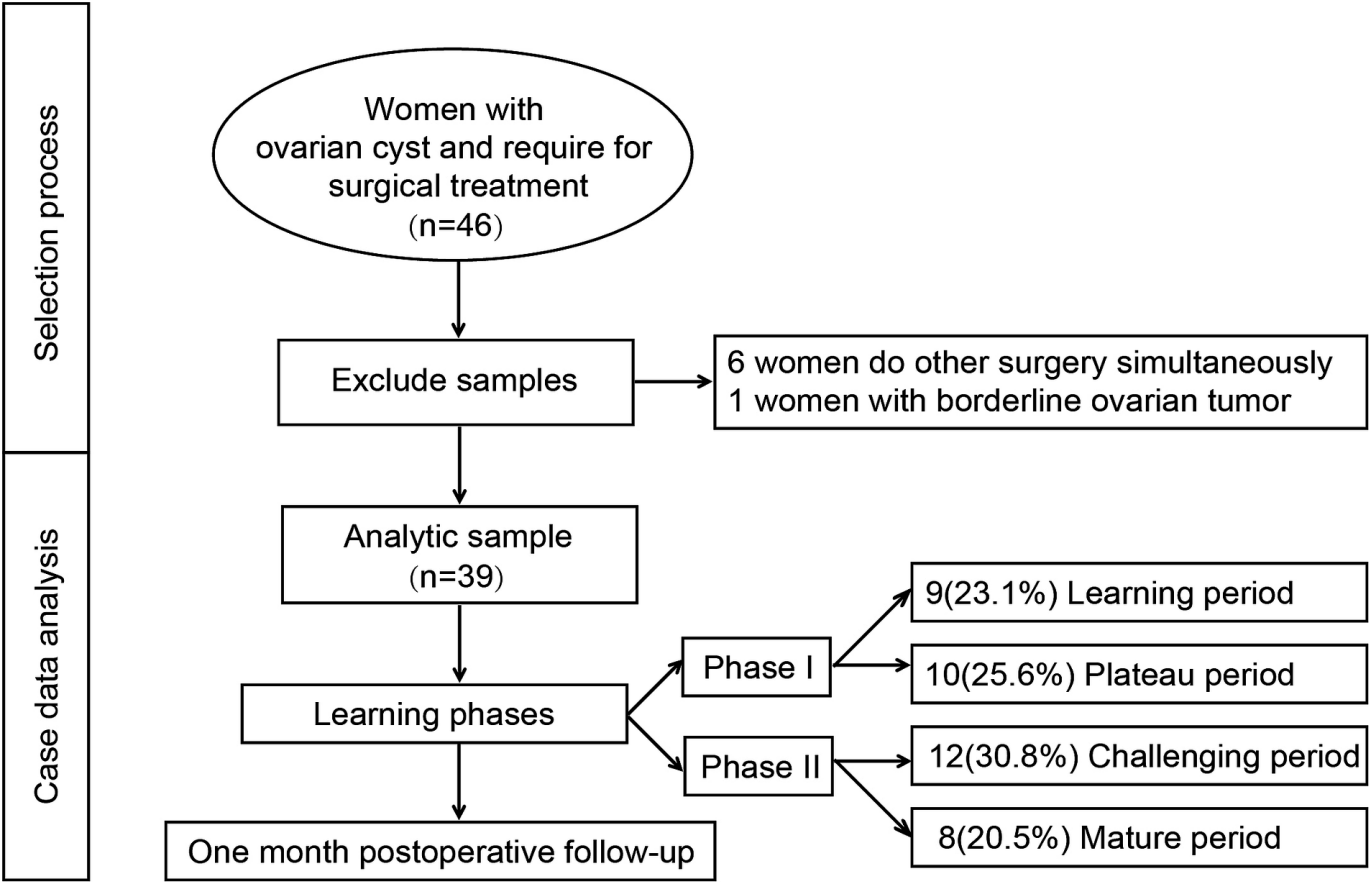
The selection process for this study.

The enrollment and exclusion process of this study is illustrated in Figure 1. A total of 46 patients meeting the inclusion criteria were initially enrolled during the study period. Six patients who underwent additional procedures intraoperatively and one patient with intraoperative frozen pathology suggestive of a borderline ovarian tumor were excluded. Ultimately, 39 patients were included in the final analysis. Among them, 19 cases were categorized into Phase I and 20 cases into Phase II. The mean age of the participants was 35.14 ± 9.73 years, and the mean operative time was 74.01 ± 30.09 min. Fifteen patients (39.5%) had a history of prior pelvic surgery, and 17 patients (43.6%) were diagnosed with endometriotic cysts based on postoperative pathology (Table 1).

**Table 1:** Description of the patients’ demographic characteristics and learning periods.

Variables	Total
Patients	39
Age (year)	35.14 ± 9.73
BMI (kg/m^2^)	22.71 ± 3.16
History of abdominal surgery	15 (39.5%)
Max diameter of cyst (cm)	6.85 ± 2.14
Bilateral ovarian cyst	11 (28.2%)
Cyst types	
Endometriotic ovarian cyst	17 (43.6%)
Non-endometriotic ovarian cyst	22 (56.4%)
Learning periods	
Learning period	9 (23.1%)
Plateau period	10 (25.6%)
Challenging period	12 (30.8%)
Mature period	8 (20.5%)

BMI, body mass index.

Intraoperative conversion to transumbilical laparoscopy occurred in three cases (7.7%), and two patients (5.1%) experienced perioperative complications—one case of rectal injury and one case of postoperative paralytic ileus. Univariate analysis revealed that, compared to Phase I, patients in Phase II had a significantly higher proportion of prior pelvic surgery, larger maximum cyst diameter, and higher incidence of intraoperative pelvic adhesions (*p* < 0.05, Table 2).

**Table 2:** Description of the patient characteristics by different learning periods.

Variables	Phase I		Phase II		*P*-value^*^
	Learning period	Plateau period	Challenging period	Mature period	
Patients	*N* = 9	*N* = 10	*N* = 12	*N* = 8	
Age (year)	34.62 ± 9.38	34.56 ± 11.47	35.38 ± 9.13	36.09 ± 8.52	0.735^a^
BMI (kg/m^2^)	22.19 ± 2.95	23.01 ± 3.50	22.56 ± 2.97	23.17 ± 3.23	0.860^a^
History of abdominal surgery	2 (22.2%)	2 (20.0%)	6 (41.7%)	5 (50.0%)	0.029^b^
Max diameter of cyst (cm)	5.74 ± 1.87	6.17 ± 1.91	7.81 ± 2.53	7.49 ± 2.04	0.017^a^
Endometriotic ovarian cyst	3 (33.3%)	4 (40.0%)	6 (50.0%)	4 (50.0%)	0.408^b^
Bilateral ovarian cyst	1 (11.1%)	2 (20.0%)	4 (33.3%)	4 (50.0%)	0.155^b^
Operative information					
Procedure time (min)	83.95 ± 24.77	64.38 ± 23.40	78.26 ± 34.84	68.51 ± 34.88	0.941^a^
Bleeding volume (ml)	69.33 ± 67.37	58.74 ± 59.33	81.11 ± 92.84	62.48 ± 65.26	0.677^a^
Pelvic adhesion	1 (11.1%)	1 (10.0%)	5 (41.7%)	3 (37.5%)	0.035^b^
Surgical conversion	1 (11.1%)	0 (0%)	1 (8.3%)	1 (12.5%)	1.000^b^
Post-operative information					
Postoperative fever	1 (11.1%)	0 (0%)	1 (8.3%)	0 (0%)	1.000^b^
Complications	0 (0%)	0 (0%)	1 (8.3%)	1 (12.5%)	0.487^b^

BMI, body mass index.

*The *p*-value refers to the comparison between Phase I and Phase II.

^a^Average and standard deviation. Student’s *t*-test.

^b^Number (percentage). Fisher Exact Test.

**Figure 2:**
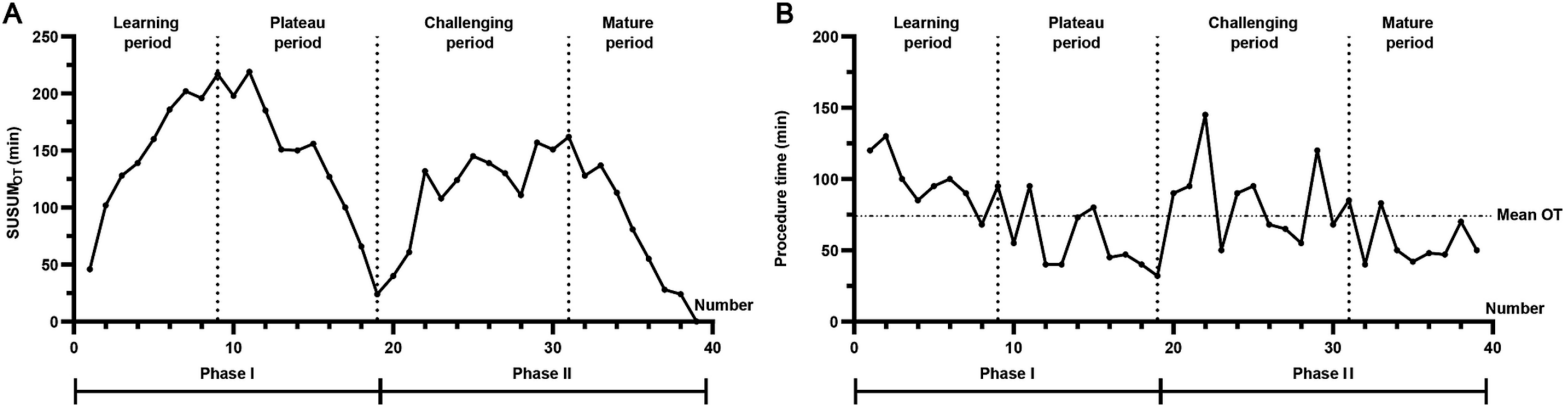
Operative times and CUSUMOT trends for each consecutive case. **(A)** According to the CUSUMOT curve, Phase I was divided into a learning period (9 cases, 23.0%) and a plateau period (10 cases, 25.6%), while Phase II was divided into a challenging period (12 cases, 30.8%) and a mature period (8 cases, 20.5%). The surgical team achieved the plateau period after completing 9 relatively simple vNOTES procedures in Phase I, indicating initial mastery of the technique. After 12 cases in Phase II, the team reached the mature period, indicating full competency in vNOTES cystectomy. **(B)** Operative times during the learning and challenging periods were slightly longer than the average, while times during the plateau and mature periods were shorter.

Operative times and CUSUMOT trends for each consecutive case were analyzed chronologically (Figure 2). According to the CUSUMOT curve, Phase I was divided into a learning period (9 cases, 23.0%) and a plateau period (10 cases, 25.6%), while Phase II was divided into a challenging period (12 cases, 30.8%) and a mature period (8 cases, 20.5%) (Figure 2A). The surgical team achieved the plateau period after completing nine relatively simple vNOTES procedures in Phase I, indicating initial mastery of the technique. After 12 cases in Phase II, the team reached the mature period, indicating full competency in vNOTES cystectomy. As shown in Figure 2B, operative times during the learning and challenging periods were slightly longer than the average, while times during the plateau and mature periods were shorter.

Based on postoperative pathological findings, 17 cases were classified as endometriotic cysts and 22 as non-endometriotic cysts. In all learning curve phases, surgeries for endometriotic cysts tended to have longer operative durations compared to non-endometriotic cysts, though the differences were not statistically significant. In Phase I, there were seven cases of endometriotic cysts (three during the learning period and four during the plateau period). In Phase II, 10 such cases were recorded (six in the challenging period and four in the mature period) (Table 3).

**Table 3:** Analysis of operative time across different learning periods based on pathological findings.

Variables		Non-endometriotic ovarian cyst (*n* = 22)	Endometriotic ovarian cyst (*n* = 17)	*P*-value
Phase I	Learning period (*n* = 9)	77.50 ± 22.89	96.85 ± 28.94	0.346
	Plateau period (*n* = 10)	58.82 ± 21.22	72.72 ± 26.64	0.407
	*P*-value	0.173	0.310	
Phase II	Challenging period (*n* = 12)	69.29 ± 25.81	87.23 ± 41.97	0.393
	Mature period (*n* = 8)	61.58 ± 22.29	75.44 ± 27.23	0.461
	*P*-value	0.629	0.605	

After adjusting for age, BMI, history of pelvic surgery, cyst type, and parity, multivariate linear regression analysis identified pelvic adhesions, learning curve phase, laterality of the cysts, and maximum cyst diameter as independent predictors of operative time. Specifically, pelvic adhesions (*β* = 6.92, 95% CI: 1.08–12.76, *p* = 0.027), learning curve phase (*β* = −17.10, 95% CI: −26.57 to −7.63, *p* = 0.035), bilateral cysts (*β* = 6.38, 95% CI: 3.34–9.42, *p* = 0.019), and cyst diameter (*β* = 2.85, 95% CI: 0.74–4.96, *p* = 0.026) were significant. For each 1 cm increase in cyst diameter, operative time increased by approximately 2.85 min. Operative times in Phase II were reduced by 17.10 min compared to Phase I. Moreover, pelvic adhesions and bilateral ovarian cysts were associated with an increase in operative time of approximately 6.9 min and 6.4 min, respectively (Table 4).

**Table 4:** Multivariate regression analysis of perioperative factors potentially associated with operative time.

Variables	Beta	95% CI	*P*-value	VIF
*R*^2^ = 0.167				
Age (year)	1.16	−0.17 ~ 2.49	0.725	1.26
BMI (kg/m^2^)	1.47	−0.91 ~ 3.85	0.263	1.21
History of abdominal surgery	3.37	−1.07 ~ 7.81	0.849	1.65
Pelvic adhesion	6.92	1.08 ~ 12.76	0.027	1.61
Learning phases	−17.10	−26.57 ~ −7.63	0.035	1.19
Bilateral ovarian cyst	6.38	3.34 ~ 9.42	0.019	1.25
Endometriotic ovarian cyst	4.22	−0.79 ~ 9.23	0.077	1.58
Max diameter of cyst (cm)	2.85	0.74 ~ 4.96	0.026	1.26

BMI, body mass index.

In total, there were two intraoperative conversions and one perioperative complication. Both cases requiring intraoperative conversion were successfully completed via transumbilical laparoscopy—one during the learning phase and one during the mature phase. The reasons for conversion were as follows: in one case, the incision was misdirected toward the posterior cervical lip, resulting in entry into the posterior uterine wall and failure to access the pelvic cavity; in the other case, severe pelvic adhesions had obliterated the rectouterine pouch. One patient experienced postoperative pelvic hemorrhage, which was successfully managed with conservative pharmacological treatment and discharged in stable condition (Table 2).

## Discussion

This study provides a comprehensive analysis of the learning curve associated with vNOTES for ovarian cystectomy. Using CUSUM analysis of operative time (CUSUMOT), we objectively delineated four distinct stages in the learning trajectory: learning, plateau, challenging, and mature phases. Our findings demonstrate that technical proficiency in vNOTES ovarian cystectomy can be achieved after approximately 20 cases, with noticeable reductions in operative time and complication rates thereafter. Moreover, we identified several independent predictors of operative time, including pelvic adhesions, cyst laterality, cyst diameter, and the surgeon’s position along the learning curve.

Consistent with existing literature on vNOTES hysterectomy and adnexectomy, our results confirm that vNOTES is a feasible and safe approach for ovarian cystectomy, even in cases with moderate surgical complexity (14, 15). However, the learning curve for vNOTES ovarian surgery appears to be more protracted compared to other gynecologic procedures due to the technical demands of enucleating cysts through a confined posterior colpotomy incision, limited triangulation, and difficulty in visualizing and mobilizing adnexal structures, especially in the presence of pelvic adhesions (16–18).

The initial learning phase was characterized by relatively straightforward cases, selected based on minimal prior pelvic surgical history, unilateral cysts, and smaller cyst diameters. These cases allowed the surgical team to become familiar with the anatomical orientation, instrument handling, and dissection techniques unique to the vNOTES approach. Following this phase, a plateau period was observed, during which operative time stabilized, suggesting consolidation of the skills necessary to safely perform the procedure. Interestingly, the subsequent challenging phase coincided with the introduction of more complex cases—such as those with endometriotic cysts, bilateral lesions, and pelvic adhesions—which resulted in a transient increase in operative time. Nevertheless, this phase was crucial for the team to develop adaptive strategies and refine their technique, ultimately leading to the mature phase, characterized by efficient, streamlined performance and minimal complications (19, 20).

Multivariate analysis revealed that pelvic adhesions and bilateral cysts were associated with an approximate increase of 6.9 and 6.4 min in operative time, respectively. These findings underscore the importance of meticulous preoperative assessment and case selection, particularly during the early phase of the learning curve (12, 18). Additionally, the observation that each 1 cm increase in cyst diameter extended the operative duration by 2.85 min highlights the need for heightened surgical precision when managing larger cysts in the vNOTES setting.

Our study also reported a low rate of intraoperative conversion (7.7%) and perioperative complications (5.1%), both of which occurred at the margins of the learning curve. One conversion during the learning phase resulted from inadvertent entry into the posterior uterine wall due to misdirected incision, and the other, during the mature phase, was due to severe pelvic adhesions obliterating the rectouterine pouch. These events emphasize that while surgical experience mitigates risk, anatomical variations and pathological complexity remain significant intraoperative challenges in vNOTES (16, 21, 22).

This study has several strengths. First, it is among the few to specifically examine the learning curve for vNOTES ovarian cystectomy with phase-based CUSUM analysis. Second, the prospective design and well-defined inclusion criteria enhance the validity of our findings. However, certain limitations must be acknowledged. The study was conducted at a single tertiary center with a dedicated surgical team, which may limit generalizability to institutions with varying levels of laparoscopic expertise. Moreover, although we excluded patients with clear preoperative evidence of malignancy, one case of borderline tumor was discovered intraoperatively, reflecting the inherent limitations of preoperative diagnostics in ovarian pathology.

In summary, our findings suggest that vNOTES is a viable and increasingly efficient approach for ovarian cystectomy as surgical experience accumulates. Structured training programs, careful patient selection, and progressive case complexity, are essential to optimizing the learning process and ensuring patient safety during the adoption of this minimally invasive technique. Future multicenter studies with larger sample sizes and long-term follow-up are warranted to validate these findings and further explore the oncologic safety and fertility outcomes associated with vNOTES in ovarian surgery.

## Conclusion

This study demonstrates that vNOTES is a safe and feasible minimally invasive approach for ovarian cystectomy, with a clearly defined learning curve. Proficiency can be achieved after approximately 20 cases, following a progression through learning, plateau, challenging, and mature phases. Operative time significantly decreases as surgical experience increases, and is independently influenced by factors such as pelvic adhesions, cyst laterality, and cyst size. These findings highlight the importance of structured training and appropriate case selection in the early learning phase to ensure surgical safety and efficiency. The use of CUSUM analysis provides an objective framework for assessing surgical competency and may serve as a valuable tool in training and credentialing programs for vNOTES procedures.

## Data Availability

The original contributions presented in the study are included in the article/supplementary material, further inquiries can be directed to the corresponding authors.
